# Researcher perspectives on ethics considerations in epigenetics: an international survey

**DOI:** 10.1186/s13148-022-01322-7

**Published:** 2022-09-02

**Authors:** Charles Dupras, Terese Knoppers, Nicole Palmour, Elisabeth Beauchamp, Stamatina Liosi, Reiner Siebert, Alison May Berner, Stephan Beck, Ian Charest, Yann Joly

**Affiliations:** 1 Centre of Genomics and Policy, McGill University, 740, Avenue Dr. Penfield, Suite 5200, Montreal, QC H3A 0G1 Canada; 2 Present Address: School of Public Health, University of Montreal, 7101 Parc avenue, Montreal, QC H3N 1X9 Canada; 3 Centre of Genomics and Policy, McGill University, 740, Avenue Dr. Penfield, Suite 5200, Montreal, QC H3A 0G1 Canada; 4 Centre for Health Ethics and Law, University of Southampton, Building 4, Highfield, Southampton, SO17 1BJ UK; 5 Institute of Human Genetics, Ulm University, Albert-Einstein-Allee 11, 89081 Ulm, Germany; 6 Barts Cancer Institute, Queen Mary University of London, Charterhouse Square, London, EC1M 5PZ UK; 7 University College London (UCL) Cancer Institute, 72 Huntley Street, London, WC1E 6BT UK; 8 Department of Psychology, Université de Montréal, 90, Avenue Vincent-d’Indy/Boulevard Édouard-Montpetit, Montréal, QC H2V 2S9 Canada

**Keywords:** Epigenetics, Ethics, ELSI, Conduct of research, Knowledge translation, Life insurance, Direct-to-consumer testing, Immigration, Forensics

## Abstract

Over the past decade, bioethicists, legal scholars and social scientists have started to investigate the potential implications of epigenetic research and technologies on medicine and society. There is growing literature discussing the most promising opportunities, as well as arising ethical, legal and social issues (ELSI). This paper explores the views of epigenetic researchers about some of these discussions. From January to March 2020, we conducted an online survey of 189 epigenetic researchers working in 31 countries. We questioned them about the scope of their field, opportunities in different areas of specialization, and ELSI in the conduct of research and knowledge translation. We also assessed their level of concern regarding four emerging non-medical applications of epigenetic testing—i.e., in life insurance, forensics, immigration and direct-to-consumer testing. Although there was strong agreement on DNA methylation, histone modifications, 3D structure of chromatin and nucleosomes being integral elements of the field, there was considerable disagreement on transcription factors, RNA interference, RNA splicing and prions. The most prevalent ELSI experienced or witnessed by respondents were in obtaining timely access to epigenetic data in existing databases, and in the communication of epigenetic findings by the media. They expressed high levels of concern regarding non-medical applications of epigenetics, echoing cautionary appraisals in the social sciences and humanities literature.

**Supplementary Information:**

The online version contains supplementary material available at 10.1186/s13148-022-01322-7.

## Introduction

The field of epigenetics sheds light on molecular mechanisms involved in the regulation of gene expression [1, 2]. Over the past decade, a growing number of bioethicists, legal scholars and social scientists have commented on the potential implications of scientific and technological developments in epigenetics on medicine and society [3–6]. Among enthusiastic appraisals, the field is believed to offer a deeper understanding of the developmental origins of health and diseases, biosocial and molecular processes through which diseases and health disparities come into being, and public policies which could contribute to preventing them [7–9]. Promising avenues also include the use of epigenetic technologies for disease prediction, diagnosis and treatment [10–12].

At the same time, ethical, legal and social issues (ELSI) have been identified, ranging from concerns about the protection of privacy of epigenetic research participants [13–15], to risks of discrimination based on individual epigenetic information [16]. ELSI may arise in the conduct of epigenetic research, where researchers may wonder which research results or incidental findings should be considered medically relevant and disclosed to study participants [17]. ELSI may also arise in the knowledge translation processes, for example, concerns have been reported about the often premature, incomplete or misleading communication of scientific findings in epigenetics by the media [18, 19].

When it comes to the risks of misuse of individual epigenetic information, policies have been adopted by many countries over the past 25 years to prevent genetic discrimination [20]. However, because these policies focus on “*genetic* characteristics” and “the results of *genetic* tests,” they don’t seem to provide a regulatory framework for the use of epigenetic information [21, 22]. This gap in oversight may be problematic considering the growing interest of multiple stakeholders in using epigenetic technologies, including life insurance companies, forensic investigators [23–27] and immigration agencies [28–30]. The rapid growth of the consumer epigenetics industry is also raising questions in terms of the scientific validity of the tests that are advertised and sold online [31], as well as the level of compliance of direct-to-consumer epigenetic testing companies with best practice standards and regulations on health technologies developed for the consumer genomics industry [32].

The question of whether epigenetic research and technologies call for new or amended policies against potential unethical use, and if so, how they should be designed, is a complex one. First, epigenetics calls for a reflection on what biological properties make genetic variants ethically sensitive (and conceptualized as worthy of legal protections) and to what extent these properties are shared by epigenetic variants [15]. Some people may perceive epigenetic variants as being “similar enough” to genetic variants to be worthy of legal protections—considering for instance correlations between specific genetic and epigenetic variants, the pre-birth programming of many epigenetic variants, and the stability of many of those alterations over a person’s lifetime. Others may argue that epigenetic variants are substantively distinct and therefore do not warrant such protections—considering for instance the plasticity and reversibility of some epigenetic variants, and the degree of control individuals are potentially able to exert over epigenetic programming [33–36].

Second, policymaking necessarily faces important conceptual and semantic challenges. Indeed, it is unclear what type of biological variants should be considered “epigenetic” variants and be regulated as such. Some people may be inclusive and consider gene expression regulating mechanisms such as transcription factors and RNA interference as falling within the scope of the field, while other people may refer only to DNA methylation and histone modifications when speaking of epigenetic variants [37, 38]. There is even uncertainty on whether DNA methylation and histone modifications should be treated as being “similarly epigenetic,” or whether their biological properties differ in such a way that the two ought not to be conflated in policymaking. As observed by Lappé and Landecker [39], and in contrast to what is often presumed [40, 41], DNA methylation does change the linear DNA sequence when it changes cytosines into methylcytosines. Therefore, from a policy interpretation perspective, DNA methylation could arguably be conceived as a genetic process and thus fall within the jurisdiction of policies against genetic discrimination. However, this would not be the case with histone modifications.

To better understand ELSI arising in epigenetics and the way they are perceived by those working in the field, we conducted an international survey of epigenetic researchers (n = 189), soliciting their opinions on the field’s scope, their experiences of ELSI in the conduct of research and knowledge translation activities, and their level of concern regarding four non-medical applications of epigenetics in development or already in use, i.e., in life insurance, direct-to-consumer testing, immigration and forensics. This study was an opportunity to put analyses of the potential implications of epigenetics—most of which have been anticipatory and speculative—to the test by having them appraised by scientists conducting epigenetic research. The goals of this study were, first, to identify areas of consensus and divergence on emerging issues across the international community of epigenetic researchers, and second, to better understand some of the underlying reasons for hopes and concerns.

## Methods

### Survey design

Informed by our previous research on the ELSI of epigenetics and complementary literature from the social sciences and humanities, we developed a mixed-method survey designed specifically for epigenetic researchers. The survey questionnaire was distributed online using the SurveyMonkey platform. It consisted of 20 questions of different formats (multiple choice, Likert scales, close-ended and open-ended), allowing for the collection of both quantitative and qualitative data. It included questions about eligibility, socio-demographics, level of expertise and areas of specialization. Research questions touched on scope, opportunities, challenges and non-medical applications of epigenetic technology.

### Recruitment

We recruited individuals with substantial past or current research experience in epigenetics. Our recruitment strategy was multimodal. First, we used a combination of convenience and purposive sampling to create a list of 1000 potential respondents based on publicly accessible lists of faculty/alumni in epigenetic research centers and organizations (e.g., the International Human Epigenome Consortium—IHEC), participants to relevant international conferences, members of editorial boards of top-tier journals in the field, and most prolific epigenetic researchers according to Google Scholar. Using a methodology adapted from Larregue et al., we identified the 40 countries from which most publications (97.3%) in epigenetics came between 2001 and 2019 [42] and paid special attention to inviting researchers working in these 40 countries. Second, we encouraged previously identified epigenetic researchers to disseminate the link to the survey through email, Twitter and other relevant networks, expanding our reach via snowball sampling.

### Data collection

From January to March 2020, we sent potential respondents an initial personalized invitation email with our survey link, followed by two reminder emails. C.D. posted the link to the survey on Twitter on three different occasions inviting colleagues to participate and/or share. These posts were re-tweeted by organizations associated with epigenetics, and researchers with international recognition in the field. T.K. also contacted persons in major epigenetic research centers based on ScienceDirect. As the population size and composition are unknown and impossible to determine with precision, the initial sample size target was 273 respondents (CL 90% with 5% error); however, time and recruitment constraints yielded a final sample size of 189 respondents (CL 90% with 6% error).

### Data analysis

#### Quantitative analysis

For each research question in the survey, we computed a proportion of the response independently for two groups (Fig. 2A). Group differences were obtained by subtracting these proportions. To assess differences in the proportion of the responses between respondent groups, we performed a series of Monte Carlo permutation tests [43, 44]. For each respondent subgroup division independently, we randomly shuffled respondent subgroup labels, computed the proportion of positive responses according to the new shuffled group labels, and subtracted those. This procedure was repeated 10,000 times to simulate the null hypothesis that group membership and proportion of the response were unrelated. To assess the significance of response differences between groups, we compared the observed proportion difference to the simulated null distribution. If the actual group difference in proportion of the response fell within the top 5% of the simulated null distributions of differences, we rejected the null hypothesis (Fig. 2B).

#### Qualitative analysis

The four questions on non-medical applications of epigenetics garnered the greatest volume of qualitative responses: 124 of the 189 researchers who completed the survey in its entirety wrote in comments regarding at least one of the four non-medical applications (99–119 comments/question). Inductive thematic analysis was conducted by T.K. using the software NVivo 12 [45, 46]. This method was chosen because the themes emerge directly from the data itself, minimizing coder bias. Thematic analysis systematically considers not only manifest content but also themes and core ideas found in the text to represent and summarize key meanings in a qualitative data set. Themes emerged both within and across the four questions and were not mutually exclusive: i.e., a single response could be attributed to several themes. Topics needed to show up in 5% of the qualitative responses for a specific question to be considered a theme (n = 5–6). N.P. then validated the coding and coding counts under each theme and some coding was modified as necessary. For the other questions that included qualitative responses but for which we obtained a smaller volume of comments (8–22 per question), thematic analysis was conducted in Microsoft Excel. Reporting of study design and results was done in light of the consolidated criteria for reporting qualitative research (COREQ) [47].

### Ethics approval

This study received a Certification of Ethical Acceptability for Research Involving Human Subjects by the McGill University Faculty of Medicine Institutional Review Board on November 11, 2019 (Project #A10-E67-19A; 19-10-034).

## Results

### Respondents

Among the 238 individuals who accessed the survey, 219 completed the eligibility questions and qualified for the questionnaire (eligibility rate = 92.0%). Thirty of them filled the questionnaire partially, and 189 in its entirety (completion rate = 86.3%).

Respondents reported working in various regions (n = 31), including Australia, Austria, Belgium, Brazil, China, Croatia, Finland, France, Hong Kong, India, Iran, Italy, Japan, Luxembourg, Mexico, Netherlands, New Zealand, Norway, Poland, Russia, Saudi Arabia, Singapore, South Korea, Spain, Sweden, Switzerland and Taiwan (Table 1A). However, 62.5% reported working in one of the top four most represented countries (the USA, Germany, Canada and the UK). Although more than half (57.1%) did not speak English as their first language, almost all respondents (97.9%) reported conducting their research in English only.

**Table 1:**
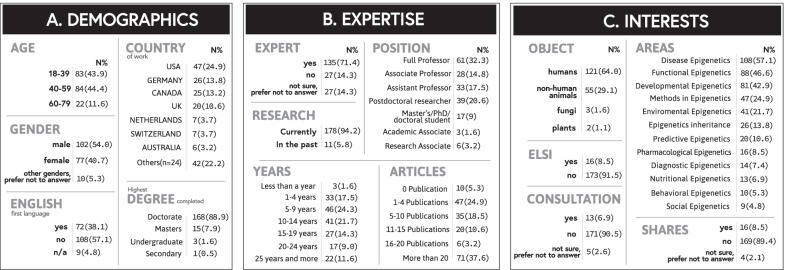
Respondent characteristics (n = 189)

The group was composed of 43.9% early-career (18–39 years old (y/o)), 44.4% mid-career (40–59 y/o) and 11.6% late-career (60–79 y/o) researchers. Fifty-four percent of participants identified as male, 40.7% as female, and 5.3% responded “other/prefer not to answer.” Respondents were highly educated, with 88.9% having completed a doctorate degree, 64.6% holding a position of Professor (Full, Associate or Assistant) and more than two-thirds (71.4%) self-identifying as experts in epigenetics (Table 1B).

Most respondents (94.2%) shared that they currently conduct research in epigenetics, while 5.8% did so in the past. Most (80.9%) relayed more than 5 years of experience conducting research in the field and 20.6% more than 20 years in the field. More than half of respondents (51.4%) stated they have published at least 10 peer-reviewed papers in the field, and 8.5% had been involved in the publication of an article related to the ethical, legal and social implications of epigenetics.

Researchers qualified their specialization most often as being within the subfields of Disease Epigenetics (57.1%), Functional Epigenetics (46.6%), Developmental Epigenetics (42.9%), Methods in Epigenetics (24.9%), and Environmental Epigenetics (21.7%) (Table 1C). Sixty-four percent of all respondents have conducted research concerning humans. A few participants (6.9%) have served as consultants for private companies commercializing epigenetic technologies, and 8.5% reported holding shares in such companies.

### Scope of the field

**Fig. 1:**
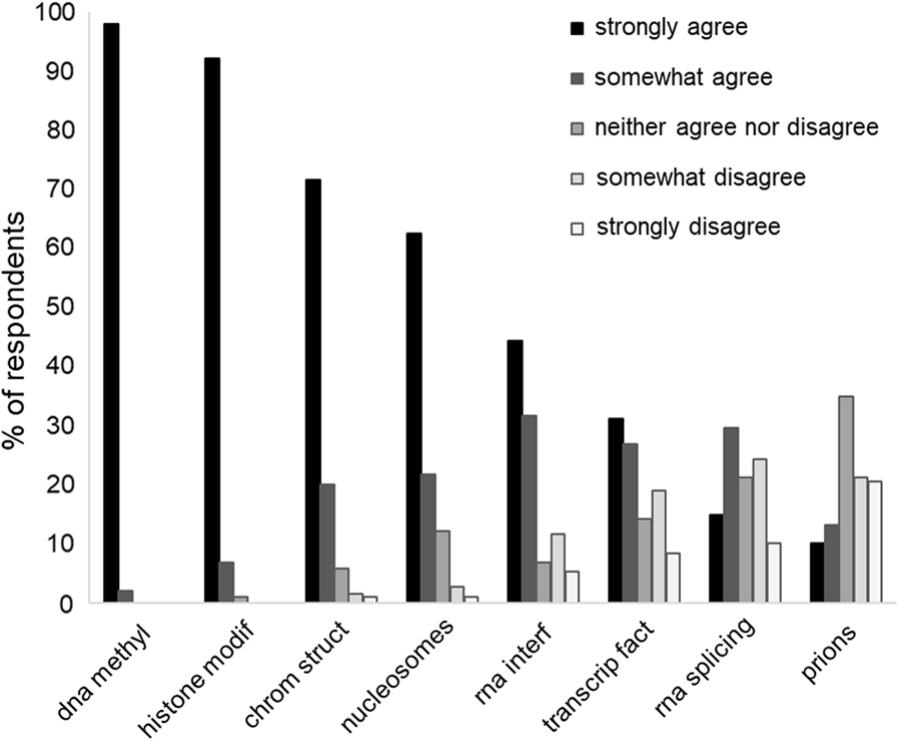
Epigenetic researchers’ perceptions of the scope of their field

The first set of research questions aimed at determining which among different types of biological variants were perceived by respondents to be included in the field of epigenetics. There was little disagreement over the inclusion of DNA methylation (0%), histone modifications (0%), the 3D structure of chromatin (2.65%) and nucleosomes (3.7%). As shown in Fig. 1, challenges inherent to defining the scope of epigenetics are best illustrated by the distribution of responses as to whether the study of interfering RNA, transcription factors, RNA splicing and prions should be considered as research in epigenetics.

**Fig. 2:**
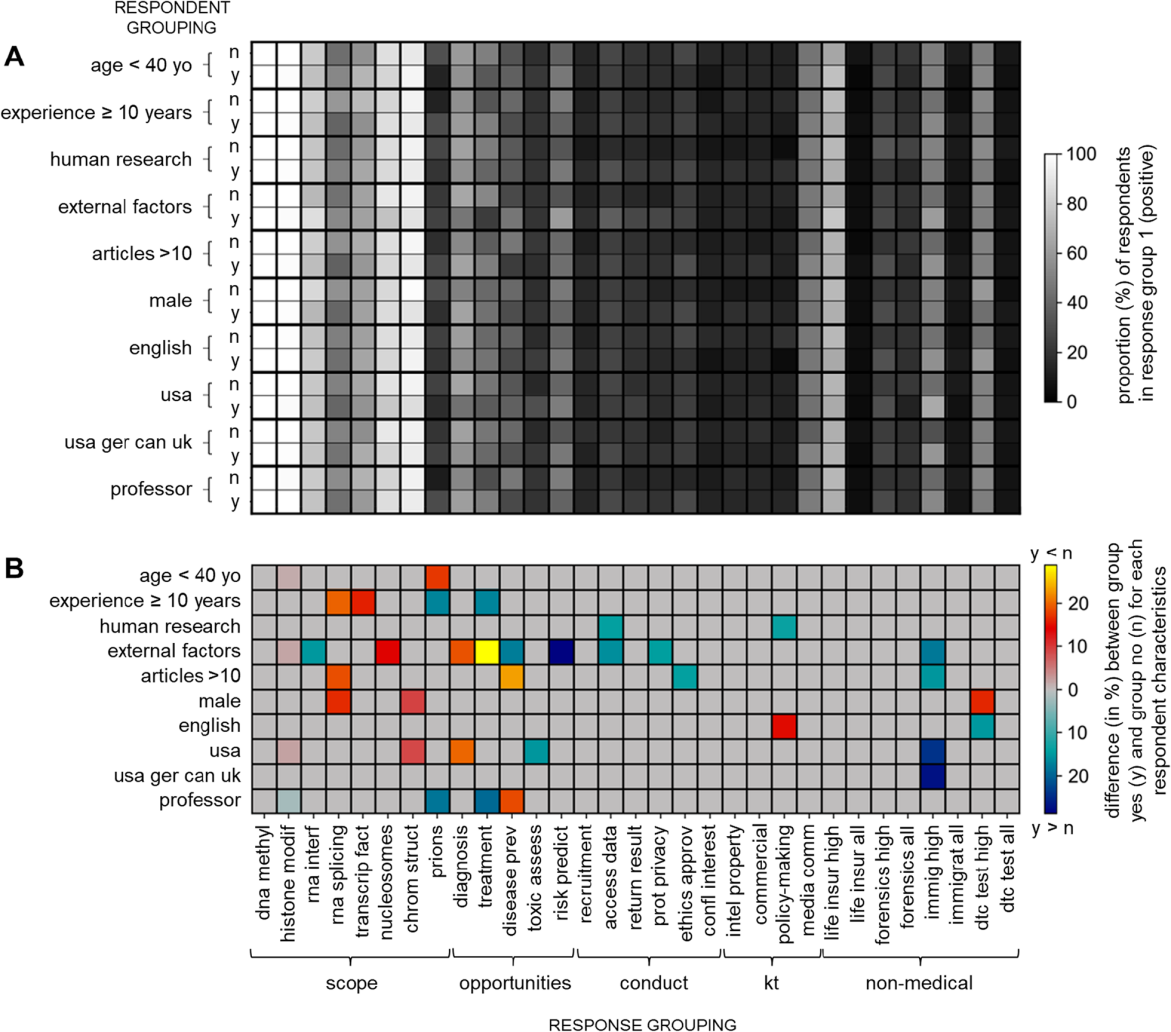
Influence of respondent characteristics on responses to research questions. **A** Proportion (%) of respondents in response group 1 (positive) for each group of respondents (see Additional file 1). **B** Difference between proportions in (**A**) (gray boxes not statistically significant)

Junior researchers appeared to have divergent opinions from their senior counterparts regarding the inclusion of transcription factors, RNA splicing, and prions (see Fig. 2). Respondents having less than 10 years of experience in the field, and those with no more than 10 peer-reviewed publications, more often believed epigenetics includes the study of RNA splicing. Similarly, respondents with less than 10 years of experience more often believed epigenetics includes the study of transcription factors. By contrast, respondents who are professors, 40 y/o or greater, and have 10 + years of experience in the field more often believed that the study of prions should be considered part of the field. Respondents who did not identify as male more often responded that epigenetics includes the study of RNA splicing and the 3D structure of chromatin, and researchers working outside of the USA were also more likely to include the latter in their field than researchers working in the USA.

### Most promising opportunities

The second set of questions in the survey asked researchers to rank the most promising medical opportunities offered by epigenetic research among five areas:

- toxicity assessment: assessing the biological effects of different products or devices, for instance in clinical trials;
- disease prevention: identifying causal factors of diseases and preventing them;
- risk prediction: anticipating future diseases;
- diagnosis: detecting diseases more accurately or at earlier stages; and,
- treatment: developing interventions that can cure or alleviate some diseases.

**Fig. 3:**
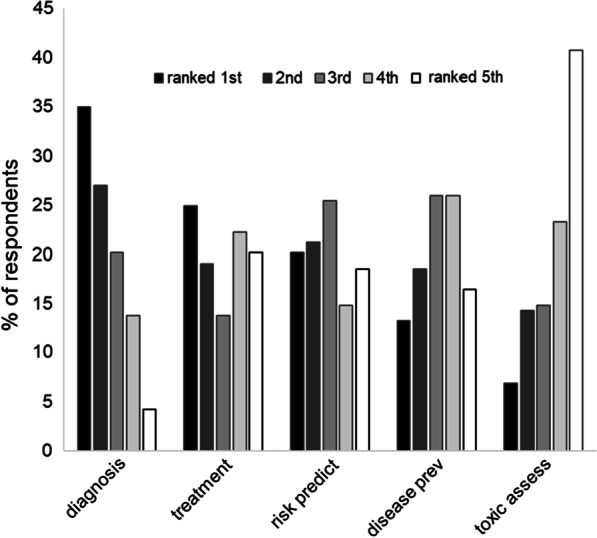
Epigenetic researchers' perceptions of the most promising areas in their field (Question: According to you, the most promising opportunities epigenetics hold are for…)

All five potential applications generated enthusiasm from a significant number of respondents, a few of them commenting that these are very challenging to rank (n = 6). However, diagnosis was preferred (ranked 1st by most respondents; 34.9%), and toxicity assessment was perceived as the least promising (ranked 5th by most respondents; 40.7%). Figure 3 presents the results of this ranking exercise.

Senior researchers (professors and respondents with 10 + years of experience) more often ranked treatment within the top 2 most promising opportunities offered by the field, whereas junior researchers were more inclined to rank disease prevention in the top 2. A similar discrepancy was observed based on the regions where respondents conduct their research. Respondents working outside of the USA more often believed diagnosis ranks in the top 2, whereas those in the USA more often believed toxicity assessment ranks in the top 2. The type of research conducted by respondents also seemed to influence their ranking. Respondents conducting their research at least in part on external factors influencing epigenetic modifications (i.e., in the subfields of nutritional, behavioral, environmental and social epigenetics) more often prioritized risk prediction and disease prevention, whereas those not conducting their research at least in part in these four subfields more often prioritized diagnosis and treatment.

### Conduct of research

**Fig. 4:**
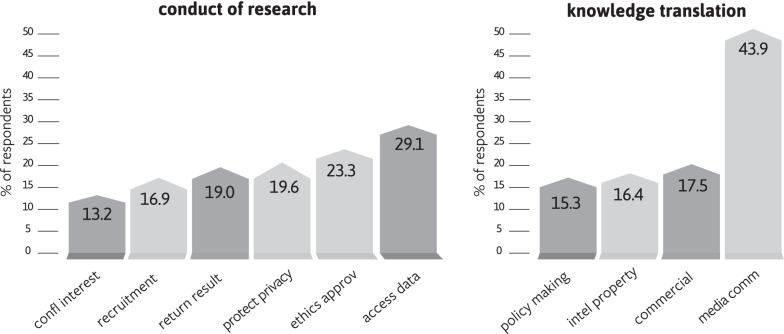
Epigenetic researchers' experiences of ethical challenges and dilemmas (faced or witnessed) in the conduct of research and knowledge translation

Regarding the conduct of research, ethical challenges or dilemmas were most frequently experienced or witnessed by respondents in relation to trying to access epigenetic data in existing databases (29.1%) and obtaining ethics approval for their research projects (23.3%) (Fig. 4). Respondents conducting research on external factors influencing epigenetic variants more often reported having experienced problems accessing epigenetic data and witnessed challenges regarding the protection of research participants’ privacy (Fig. 2). Overall, quantitative findings and qualitative comments (n = 7) suggest that there are practical barriers to reaching a productive balance between privacy protection and research advancement. Researchers observed that privacy concerns come about because: *“epigenetic data can be equally ‘identifiable’ as genetic data, and therefore there can be ethical concerns about making the data publicly available”* (P39) and that policies mandating data sharing and depositing in publicly accessible databases may impede participant recruitment by *“making participants less willing to participate given we need to ask if we can share their data in this way”* (P111). At the same time, *“overstatement of (epi)genetic determinism or personal identifiability can place barriers due to overvaluing of the data”* (P71). Some researchers added that the challenges in reaching this balance are compounded by both practical and perceived overlap between the fields of genetics and epigenetics and shared several examples (n = 5): *“justifiably or not, participants and ethics boards cannot tell it [epigenetic data] apart from other genetic data”* (P71); *“it was challenging to discuss epigenetics with our ethics board on a study that had collected samples for genetic testing in an earlier era where we did not foresee that the DNA could also tell us about recent human behavior beyond the genes”* (P155). In the words of one respondent: *“We need to find a better balance between protecting against perceived threats and enabling research. […] We need to work together to find a solution”* (P178).

### Knowledge translation

The communication of scientific findings in epigenetics by the media was the issue that generated adversarial experiences for the highest proportion of respondents (43.9%) (Fig. 4). Some expanded on the issue (n = 20), describing communication as often “inaccurate,” “exaggerated,” “premature” and/or “misleading.” Respondents attributed this to three factors. First, *“epigenetics is a complex topic that requires some biology background to understand. It’s difficult to convey these concepts to the lay population and journalists to engage them in the research”* (P34). Second, because the media have commercial interests in vetting and framing the information to attract readers, communication of findings is *“often way over-hyped and suggests biological links that are not validated by data”* (P116). Third, some felt that researchers and research institutions could be more rigorous in their communication to the media and avoid *“over-claiming of significance of studies in press releases by scientific institutions”* (P101). Exaggerated statements of individual control over epigenetic variants, and transgenerational epigenetic inheritance, were most frequently reported. Respondents also criticized the instrumentalization of hype surrounding epigenetics for commercialization purposes, such as the marketing of cosmetics (n = 5).

### Non-medical applications

**Fig. 5:**
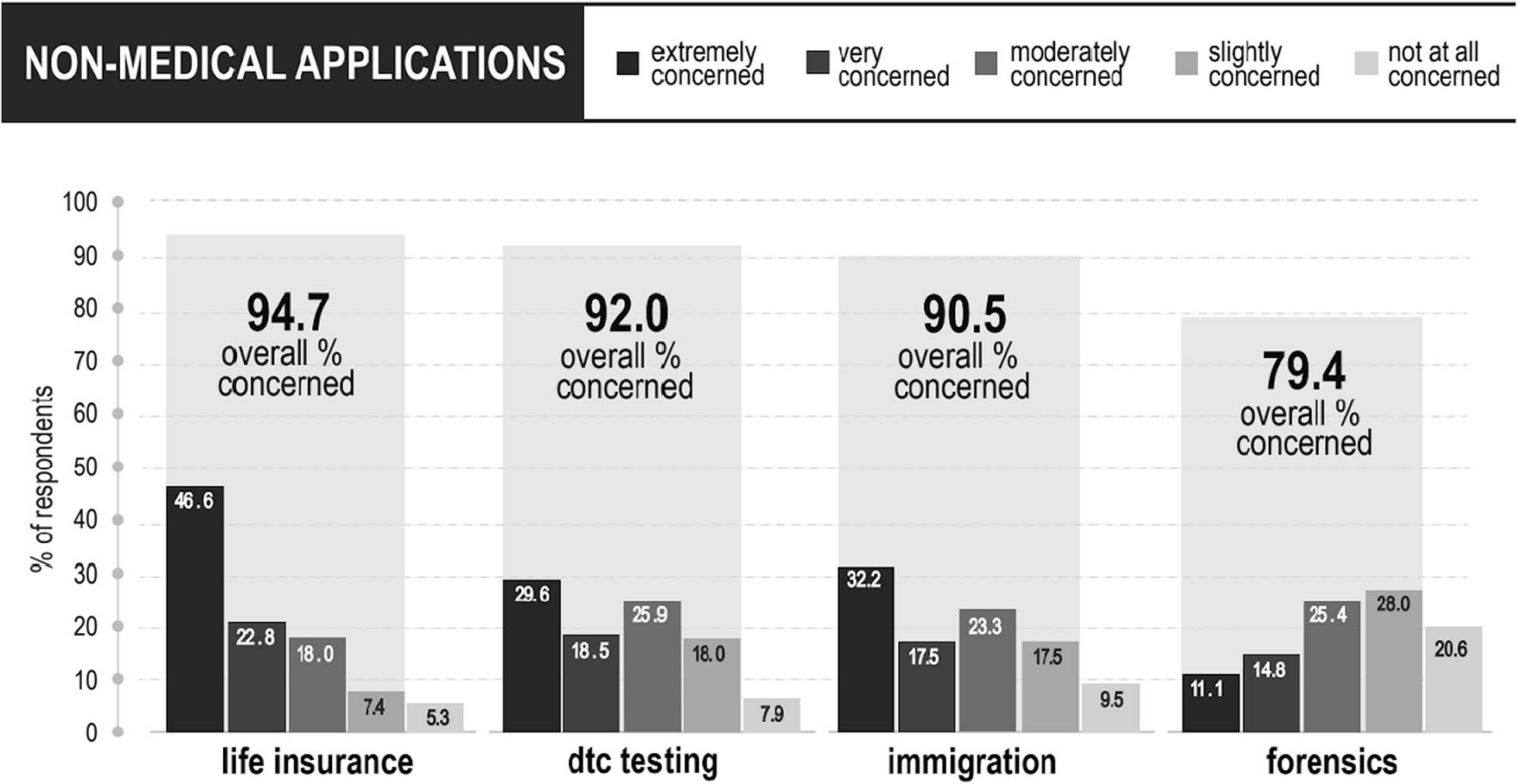
Epigenetic researchers’ level of concern regarding non-medical applications of findings in their field

As shown in Fig. 5, most researchers were concerned by emerging non-medical applications of their field. Levels of concern varied across respondents and across the four applications. A high proportion reported being either “very” or “extremely” concerned regarding the potential use of epigenetic tests in insurance (69.4%), immigration (49.8%) and by direct-to-consumer testing companies (48.1%). Forensic applications generated less concern, with only 25.9% of respondents being either “very” or “extremely” concerned and almost half (48.6%) either “not at all concerned” or only “slightly concerned”.

### Life insurance

Life insurance was the most unpopular non-medical application among survey respondents, with 94.7% being concerned to some extent, including more than two-thirds (69.3%) being “very” (22.7%) or “extremely” (46.6%) concerned. This application also garnered the most qualitative comments: 119 in total. Many researchers asserted that the science is not sufficiently understood, reliable or validated for such an application (n = 63): *The error on the epigenetic clocks is quite large and the quality control of methylation microarrays in general is quite poor. While the properties of the Horvath estimator apply to populations, they are still too crude to apply to individual*s (P155). There were also observations (n = 12) relating to the interplay between the application and the *“nature”* of epigenetics: e.g., that some epigenetic variants are plastic and may change over the life course and can be influenced by sociocultural and physical environments. For instance, one respondent argued: *“we know that epigenetics can be altered so how can an assessment be made based on one time point. Also, other factors impact health and lifespan like genetics, diet, etc.”* (P136).

There was also a widely shared sentiment that it might be ethically questionable for life insurance companies to factor in epigenetic information within their operations (n = 67). Some researchers made parallels with the use of genetic information (n = 20): *“I don't think insurance companies should have access to epigenetic/genetic data”* (P213); *“factors that are predetermined at birth, including [some] epigenetic markers, should not be used for financial risk assessment”* (P47). Others argued that it is against the very purpose of insurance companies (n = 6), or as one respondent explained: because it *“contradicts the idea of an insurance (balancing personal risks in community)”* (P123). A significant number of respondents (n = 50) commented regarding potential differential treatment or profiling based on epigenetic characteristics. One researcher wrote: *“Every person should have the same insurance opportunities, regardless of their epigenetic status”* (P59) and another more strongly:

*Discrimination in the valuation of life, while financially logical for an underwriting institution, is fundamentally unjust, regardless of how precise the prediction. And such predictions will merely recapitulate existing contributors to lifespan - ones that are often not allowed to be discriminated against such as race/ethnicity*. (P71).

Within the risk of discrimination, some researchers were worried about rising insurance costs (n = 12) and impeded access (n = 10) that would disproportionately impact those who are already systemically disadvantaged (n = 8): *“It could lead to persons not being accepted by insurances anymore or paying extremely high fees”* (P122). These risks led to discussions of legislation and regulation regarding the potential uses of epigenetic tests in life insurance (n = 9), with some respondents urging life insurance companies to follow similar laws and guidelines as they do for genetics (n = 5) and others interestingly, being “not at all” or only “slightly” concerned because they are confident legal protections will be put in place to prevent misuse (n = 4).

### Direct-to-consumer testing

The vast majority of respondents were concerned about the emergence of direct-to-consumer epigenetic testing for health and wellbeing (92.1%). In terms of distribution of concern, about half (48.2%) were “very” or “extremely” concerned and a quarter only “slightly” or “not at all” concerned (25.9%). Those who did not identify as male (i.e., those who identified as female, another gender or preferred not answer) were more often “very” or “extremely concerned” than male respondents. There were 102 comments regarding this application. Researchers questioned the validity and actionability of the tests being advertised and sold online (n = 51), with some expressing concerns that direct-to-consumer epigenetic testing companies are overstating or misrepresenting science for profit (n = 33): *“There are no overtly actionable epigenetic marks that can be used”* (P164); *“A lot of smoke and mirrors. The science is not there yet”* (P197). There were some apprehensions that consumer epigenetics could affect the reputation of the field (n = 5); *“my concern is that it makes the science as a whole seem less credible if there are people using it in a non-rigorous way and it would be a real shame if people don't recognize the more serious potential benefits of epigenetic research”* (P35). Respondents also had misgivings regarding how the public would understand, react to, and act upon the results of such tests (n = 30): *“Very few consumers could properly interpret the results and I don't trust the companies to properly explain the data”* (P27). It was suggested that companies be required to educate potential customers on the risks and limitations of their products (n = 8) and there were calls for medical expert delivery of results (n = 6). There were also comments pertaining to consumer privacy and possible data misuse (n = 15). Some noted *“serious data privacy concerns, from companies potentially selling their data, to data security”* (P37), and feared that *“the information might end up being used for other purposes without the consumers’ permission”* (P184). At the same time, other researchers emphasized that respecting the autonomy of consumers was important (n = 13): *“there’s a lot of other similar nonsense out there. In the end, it’s an individual choice unlike health insurance etc…”* (P127) and argued that the tests are minimal risk in what they reveal and recommend (n = 10): *“not sure what kind of action can be taken by consumers that they wouldn’t already have. If they have an unhealthy lifestyle they will test older, maybe this will spur them into adopting a healthy lifestyle”* (P203).

### Immigration

A high percentage (90.5%) of researchers were concerned about the potential use of epigenetic age testing in immigration to confirm the minor status of undocumented asylum-seekers. The range of concern was quite similar to DTC testing with about half (49.7%) “very” or “extremely” concerned and just over a fourth (27.0%) only “slightly” or “not at all” concerned. Interestingly, researchers with more than 10 peer-reviewed publications, as well as those working in the USA, or on external factors influencing epigenetic modifications, were more likely to be “very” or “extremely” concerned about such an application. This question had 99 qualitative responses. From a science perspective, researchers pointed out that the tests are not currently accurate or reliable enough for this application (n = 42): *“epigenetic clocks don’t perform well and have the highest variance on pediatric samples”* (P151). Scientific concerns tied into worries about potential environmental (n = 11) and racial/ethnic discrimination (n = 8). They noted that the biological age of asylum-seekers may be higher than their chronological ages due to a history of harsh life conditions (stress, violence, pollution exposures): *“we know that environmental stress can make biological age seem older and being predicted as older would have serious implications on how the human being trying to migrate will be treated”* (P35). Respondents were also concerned about existing epigenetic clocks being based primarily on European-ancestry samples and not directly transposable onto many asylum-seekers: *“data based on small sample sizes and narrow ethnic population background, lack of understanding of the impact of ethnicity on such metrics”* (P67). Some viewed this potential application as particularly problematic (n = 13): *“the idea of exploiting this technology to turn people in need away is horrifying”* (P90); *“we should trust and help people instead of finding a reason to push them away”* (P81). Free consent also emerged as a potential issue (n = 5) since asylum-seekers may be put in a situation where they cannot refuse to test. In contrast, other respondents stressed that epigenetic tests may be useful evidence for immigration (n = 8): *“the access to biological age of the refugees is a logical choice for the host country”* (P171); ​​ “*it´s very important to collect all biological information to strengthen the law”* (P122).

### Forensics

The potential use of epigenetic information to refine criminal profiles generated the least concern of the four applications. Nearly half of respondents (n = 48.7%) were either “not at all” or “slightly” concerned. There were 101 qualitative comments. As was the case for the other three applications, a significant number of respondents asserted that epigenetic testing technologies are not precise and reliable enough to be used for this application at present (n = 49). Nevertheless, some researchers expressed being in favor of such a potential application considering it could help solve crimes (n = 23): *“this is why [investigators] perform forensic analysis—to identify a suspect. More information can only be helpful in this case. That said—the majority of what is stated above is not currently possible and likely will not be in our lifetimes…”* (P178). Interestingly, almost half (n = 10) of these respondents explained that they would want epigenetics to be used to help investigations, but not exclusively relied upon for convictions. As one researcher stated:

*“If it is purely to establish a profile to search for the suspect from a pool that may not be a bad approach. Having said that, experts in the field of epigenetics should be both conducting and reporting the test results. Additionally, this line of evidence should be only part of the crime-solving arsenal and not the sole basis of conviction”* (P199).

Some researchers (n = 5) also argued that since forensic investigators already have access to genetic information, the addition of epigenetic information is *“not much more”* (P189).

The most common legal concern regarding forensic applications was the potential wrongful conviction of innocent individuals (n = 18). False positives are possible, some argued, both because epigenetics as a field is not mature enough, and because of human factors: *“standards are currently way too low in the forensic field, and human error is rampant”* (P153). There was some trepidation about potential misuse by law enforcement (n = 6): *“the balance of power between the great machine of law enforcement on the one hand and individual suspects on the other, is already very unfair. Law enforcement has, all over the world, repeatedly demonstrated that they will abuse the powerful tools given to them”* (P124). Some respondents were concerned specifically about privacy (n = 12). They wrote: *“data regarding innocent people may be made public”* (P217) and this could represent *“an infringement on socio-economic status and lifestyle”* (P192).

## Discussion

Epigenetic researchers differed in their assessment of the scope of their field. While biological mechanisms related to the DNA level—such as DNA interaction and 3D packaging changes—were widely considered as epigenetic, there was considerable disagreement regarding the inclusion of mechanisms acting on the RNA and protein levels—such as interfering RNA, RNA splicing, transcription factors, and prions. As mentioned at the outset of this paper, lack of consensus on the scope of the field could have practical implications for policymaking. Down the road, it could also create uncertainty where policy interpretation is required. If legislation was to be enacted or amended by countries or regions to prevent discrimination based on the results of *epigenetic* testing specifically, in insurance or employment, for instance, it would be crucial that clear definitions of “epigenetic” be included in the law. Otherwise, questions such as whether interfering RNA testing is permitted under those laws would remain open to subjective and variable interpretations [48–50]. Disagreement over the inclusion of non-core mechanisms can also impact communication among researchers and contribute to further public confusion about the field.

As a group, researchers ranked diagnosis and treatment as the two most promising avenues for epigenetics. However, it is worth noting that junior researchers and those working on the determinants of epigenetic changes (i.e., in nutritional, behavioral, environmental, or social epigenetics) ranked prophylactic (prevention and prediction) opportunities higher. The latter may result from confirmation bias on both sides, where the specific expertise community either has an influence on the researchers’ expectations toward epigenetics, or their preexisting attitude and preferences are reflected through the selection of their research stream within the field. The fact that senior researchers tend to favor clinical applications of epigenetics (diagnosis and treatment) may be due to their level of experience with the various methodological challenges inherent in studies of the factors influencing epigenetic programming and health (e.g., dealing with multiple confounding variables, limited scientific validity of in vitro and non-human animal models in the study of complex system interactions, and replicability issues).

Regarding ELSI in the conduct of research, many respondents indicated that gaining access to epigenetic data in existing databases and obtaining ethics approval for research projects can be particularly challenging. Some of these challenges are administrative in the form of researcher and protocol vetting by data access committees. One option to address challenges related to access to biorepositories is via harmonized data governance (https://www.ga4gh.org/genomic-data-toolkit/regulatory-ethics-toolkit/, https://www.eu-stands4pm.eu/data_access) [51, 52]. The Global Alliance for Genomics and Health (GA4GH) has developed harmonized governance tools for the context of genomics that could be adapted for epigenomic research [53]. Other challenging ethical issues for researchers and research ethics boards may arise from working with consent documents for sample and data access that are not formulated broadly enough and do not permit epigenetic research using data from genetic repositories, or anonymized databases that do not allow for recontacting participants to seek their consent [54]. There have been adjustments in ethical evaluations and approaches such that broad consent for biorepositories has gained ethical acceptance [55].

Regarding ELSI in knowledge translation activities, media communication was seen by far as the most important challenge. This result echoes literature in the social sciences and humanities expressing concern about inflated understandings of individual control over one’s body, and of transgenerational epigenetic inheritance, both of which have had ripple effects in public discourse around moral responsibility for health, notably, in marketing messages publicizing new direct-to-consumer epigenetic products [19, 32, 35, 36, 56]. The field’s complexity, overstatements of research results in publications, the challenge to convey nuance and uncertainty in captivating headlines, and the tendency for information in the media to be oversimplified, were all identified as contributing to the problem [18, 19, 35, 36, 49, 56]. Respondents in this study identified both journalists and researchers as key actors in the reduction in misleading and inaccurate claims about findings in epigenetics. While the other knowledge translation activities garnered much less qualitative attention by respondents than non-medical applications, quantitative results indicate they experienced or witnessed ethical challenges or dilemmas in the commercialization of epigenetic tests or products, in relation to intellectual property, and in policymaking. Further studies will be necessary to better understand the specific reasons why these activities appear problematic to some researchers.

Regarding the four potential non-medical applications of epigenetics discussed in this paper, researchers consistently stressed that epigenetics is at a nascent stage and is not reliable or accurate enough for such uses. In the future, however, some epigenetic tests could become robust enough to allow for their use, at least from a scientific and technical perspective. For instance, epigenetic age tests may become more precise, and their accuracy may be validated in various populations. This is currently not the case and may lead to inaccurate determinations of age in highly sensitive contexts such as in the context of forensics or in the case of migrants seeking asylum under some countries’ facilitated process for minors. At the same time, it is important to keep in mind that the availability of high-performing technology does not necessarily entail the ethical and social acceptability of the different ways they might be used. Although it should always take the changing status of science into account, ethics is an endeavor of its own. As many respondents seemed to believe, what is possible is not necessarily desirable.

Our study has some limitations which it is important to keep in mind when interpreting the results. For instance, the final sample is skewed toward the USA, Germany, Canada and the UK. This is most likely due to our recruitment strategy of sending invitations to researchers in the top countries producing epigenetic research, in English, from 2001 to 2019, and in roughly proportionate numbers. For reference, those four countries produced 53.3% of publications during that period. However, researchers from other most prolific countries - China, Japan, and Italy in particular - had lower response rates than anticipated. As snowball sampling was another of our key means of recruitment, the demographics of our sample may also have been influenced by our closer professional networks being composed in large part by researchers from Canada, the USA, and Western Europe. Another possible source of selection bias is that most researchers who accepted to participate in our survey may be interested, at least to some extent, in ethics considerations related to developments in their field—in contrast to those who refused to participate. This may have inflated the group’s level of concern regarding the four non-medical applications of epigenetics, for example. Finally, it is crucial to remember that the views expressed in this paper are those of researchers. Other stakeholders may have a very different opinion on the matters explored in this study.

## Conclusion

To our knowledge, this is the first large-scale mixed-method survey exploring the critical views of epigenetic researchers on their field and its applications. In the coming years, epigenetics will undoubtedly continue to develop, and hopefully, some of its promises for medicine and public health will materialize. While this progress continues, it is important for researchers to keep reflecting on their discipline. As some respondents aptly noted, how scientific findings are communicated to the public as well as how they are used in practice can affect the conduct and reputation of science itself. In the long term, misrepresentations or controversial applications of epigenetics can contribute to distort public understandings of the field and, more generally, undermine public trust toward researchers.

Interdisciplinary and intersectoral dialogue involving epigenetic researchers are instrumental to delving further into the study of ELSI in epigenetics, and where needed, developing policies that address these emerging issues. Exploring the views of other stakeholders, such as research participants, patients, consumers, members of the industry and policymakers, will be important as well. Informed regulation and ethics frameworks for epigenetics ought not to be based solely on the views of researchers—neither should they be based solely on the views of bioethicists, social scientists, or legal scholars. A plurality of actors should be consulted, and their views considered. This study is only one step forward in better understanding perceptions of epigenetics. We hope that it will invite additional empirical work and promote discussions on the potential implications of the field for society.

## Supplementary Information

**Additional file 1:** Survey questionnaire and grouping.

## Data Availability

The data that support the findings of this study are available on request from the corresponding author, CD. The data are not publicly available due to their containing information that could compromise the privacy of research participants.
