# Supplementary material for: Researcher perspectives on ethics considerations in epigenetics: an international survey
Source: Clin Epigenetics. 2022 Sep 2;14:110. doi: 10.1186/s13148-022-01322-7 (PMC9440515; doi:10.1186/s13148-022-01322-7)
Supplement: Supplementary file 1 — Additional file 1: Survey questionnaire and grouping. [file 13148_2022_1322_MOESM1_ESM.docx]

**Supplementary Materials**

**Survey Questionnaire**

**RESPONDENT CHARACTERISTICS**

**Demographics**

AGE: How old are you?

GENDER: What gender do you identify with?

ENGLISH: What is your first language (native language)?

COUNTRY: What country do you currently do epigenetic research in (country of work/residence)?

DEGREE: What is the highest education degree you have completed?

**Expertise**

EXPERT: Would you identify yourself as an expert in epigenetics?

RESEARCH: Do you currently do research in epigenetics?

YEARS: How many years (approximately) have you done research in epigenetics?

POSITION: What is your current position?

ARTICLES: How many peer-reviewed articles (approximately) have you published on epigenetics?

**Interests**

OBJECT: What is/was the main subject of your research in epigenetics?

ELSI: Have you ever published a peer-reviewed article addressing the (potential) ethical, legal and social implications of epigenetics – by yourself or as a result of a collaboration with one or more scholar(s) from the social sciences and humanities (e.g. ethicist, medical anthropologist)?

CONSULTATION: Are you currently involved as a consultant for one or more companies commercializing epigenetic products?

AREAS: What are the main areas of your research in epigenetics? (Please select at least one and a maximum of three)

SHARES: Do you currently hold shares in one or more companies commercializing epigenetic products?

**RESEARCH QUESTIONS**

**Scope**

Epigenetics include the study of …

- DNA methylation
- Histone modifications
- Interfering RNA
- RNA splicing
- Transcription factors
- Nucleosomes
- The 3D structure of chromatin
- Prions

**Opportunities**

According to you, the most promising opportunities epigenetics hold are for: (Please rank from 1-5, 1 being the most promising and 5 the least):

- Diagnosis (i.e., to detect some diseases more accurately or at earlier stages)
- Treatment (i.e., to develop interventions that can cure or alleviate some diseases)
- Disease prevention (i.e., to identify causal factors of diseases and prevent them)
- Toxicity assessment (i.e., to assess the biological effects of different products or devices, for instance in clinical trials)
- Risk prediction (i.e., to anticipate future diseases)

**Conduct of research**

Have you experienced or witnessed ethical challenges/dilemmas in any of the following aspects of the conduct of research in epigenetics?

- Recruiting participants
- Accessing epigenetic data in existing databases
- Returning results or incidental findings to participants
- Protecting data and the privacy of research participants
- Obtaining ethics board approval
- Conflict of interest or other contextual factors that you believe may impede scientifically sound and/or ethical research in epigenetics

**Knowledge Translation**

Have you ever experienced or witnessed ethical challenges/dilemmas related to the application of findings in epigenetics?

- Regarding intellectual property or patenting
- Regarding the commercialization process
- Regarding the policy-making process
- Regarding the communication of scientific findings by the media

**Non-medical applications**

Insurance: YouSurance is the first company to use epigenetic biomarkers to assess life insurance applicants’ health and lifespan. Are you concerned about insurance companies making such uses of the findings of epigenetic research?

Forensic investigation: It has been noted that it may now be possible to produce a profile of a criminal suspect out of a drop of blood that provides details regarding age, diet, smoking status, medications, polluted environment the suspect lives in, and if they have a traumatic history of abuse. Are you concerned with epigenetic knowledge being used for forensic investigations?

Immigration: Findings from epigenetic research regarding “biological age” may be used by immigration agencies to prove the age of undocumented minor migrants seeking asylum. Are you concerned with epigenetic findings being used in immigration?

Direct-to-consumer epigenetic testing: Private companies have started offering online direct-to-consumer epigenetic testing for a variety of conditions related to health and well-being, such as biological aging, smoke exposure, and skin type. Most of these companies provide consumers with health and lifestyle advice based on their epigenetic profile. Are you concerned with epigenetics tests being offered directly to the public by private companies?

**Grouping**

**RESPONDENT GROUPING**

|  | **Group “no” (n)** | **Group “yes” (y)** |
| --- | --- | --- |
| age (age < 40 y/o) | is 40 years old or more | is under 40 years old |
| experience (experience ≥ 10 years) | has done epigenetic research for less than 10 years | has done epigenetic research for 10 years or more |
| human research | does epigenetic research only on non-human animals, fungi (e.g., yeasts), and not on humans | does epigenetic research on humans |
| external factors | does not do research in nutritional, behavioral, environmental or social epigenetics | does research in nutritional, behavioral, environmental or social epigenetics |
| articles > 10 | has published 10 or less peer-reviewed articles in epigenetcs | has published more than 10 or peer-reviewed articles in epigenetcs |
| gender (male) | does not self-identify as male | self-identifies as male |
| english | does not have english as first (native) language | does not have english as first (native) language |
| usa | does epigenetic research in a country or region other than the usa | does epigenetic research in the usa |
| usa ger can uk | does epigenetic research in a country other than the usa, germany, canada or the united kingdom | does epigenetic research in the usa, germany, canada, or the united kingdom |
| professor | is not a professor | is a professor (i.e., assistant, associate, or full professor) |

**RESPONSE GROUPING**

|  |  | **Group 0 (negative)** | **Group 1 (positive)** |
| --- | --- | --- | --- |
| scope  (inclusion in the field) | dna methyl | disagreed (somewhat or strongly) | agreed (somewhat or strongly) |
|  | histone modif | disagreed (somewhat or strongly) | agreed (somewhat or strongly) |
|  | rna interf | disagreed (somewhat or strongly) | agreed (somewhat or strongly) |
|  | rna splicing | disagreed (somewhat or strongly) | agreed (somewhat or strongly) |
|  | transcrip fact | disagreed (somewhat or strongly) | agreed (somewhat or strongly) |
|  | nucleosomes | disagreed (somewhat or strongly) | agreed (somewhat or strongly) |
|  | chrom struct | disagreed (somewhat or strongly) | agreed (somewhat or strongly) |
|  | prions | disagreed (somewhat or strongly) | agreed (somewhat or strongly) |
| opportunities | diagnosis | ranked as one of the last 3 | ranked in top 2 |
|  | treatment | ranked as one of the last 3 | ranked in top 2 |
|  | disease prev | ranked as one of the last 3 | ranked in top 2 |
|  | toxic assess | ranked as one of the last 3 | ranked in top 2 |
|  | risk pred | ranked as one of the last 3 | ranked in top 2 |
| conduct of research  (ethics challenges or issues) | recruitment | has not witnessed or experienced | has witnessed or experienced |
|  | access data | has not witnessed or experienced | has witnessed or experienced |
|  | return result | has not witnessed or experienced | has witnessed or experienced |
|  | prot privacy | has not witnessed or experienced | has witnessed or experienced |
|  | ethics approv | has not witnessed or experienced | has witnessed or experienced |
|  | confl interest | has not witnessed or experienced | has witnessed or experienced |
| knowledge translation  (ethics challenges or issues) | intel property | has not witnessed or experienced | has witnessed or experienced |
|  | commercial | has not witnessed or experienced | has witnessed or experienced |
|  | policy making | has not witnessed or experienced | has witnessed or experienced |
|  | media comm | has not witnessed or experienced | has witnessed or experienced |
| non-medical applications  (level of concern) | life insur high | is not very or extremely concerned about the (potential) use of epigenetic testing in life insurance | is very or extremely concerned about the (potential) use of epigenetic testing in life insurance |
|  | life insur all | is not concerned at all about the (potential) use of epigenetic testing in life insurance | is concerned (slightly, moderately, very or extremely) about the (potential) use of epigenetic testing in life insurance |
|  | forensics high | is not very or extremely concerned about the (potential) use of epigenetic testing in forensics | is very or extremely concerned about the (potential) use of epigenetic testing in forensics |
|  | forensics all | is not concerned at all about the (potential) use of epigenetic testing in forensics | is concerned (slightly, moderately, very or extremely) about the (potential) use of epigenetic testing in forensics |
|  | immigrat high | is not very or extremely concerned about the (potential) use of epigenetic testing in immigration | is very or extremely concerned about the (potential) use of epigenetic testing in immigration |
|  | immigrat all | is not concerned at all about the (potential) use of epigenetic testing in immigration | is concerned (slightly, moderately, very or extremely) about the (potential) use of epigenetic testing in immigration |
|  | dtc high | is very or extremely concerned about direct-to-consumer epigenetic testing | is very or extremely concerned about direct-to-consumer epigenetic testing |
|  | dtc all | is not concerned at all about direct-to-consumer epigenetic testing | is concerned (slightly, moderately, very or extremely) about direct-to-consumer epigenetic testing |
